# Effect of Standardized *Hydrangea serrata* (Thunb.) Ser. Leaves Extract on Body Weight and Body Fat Reduction in Overweight or Obese Humans: A Randomized Double-Blind Placebo-Controlled Study

**DOI:** 10.3390/nu14010208

**Published:** 2022-01-03

**Authors:** Hee-Soo Han, Kyung-Sook Chung, Yu-Kyong Shin, Jae-Sik Yu, Seung-Hyun Kang, Sun-Hee Lee, Kyung-Tae Lee

**Affiliations:** 1 Department of Pharmaceutical Biochemistry, College of Pharmacy, Kyung Hee University, Seoul 02447, Korea; heesu3620@khu.ac.kr (H.-S.H.); adella76@hanmail.net (K.-S.C.); 2 Department of Life and Nanopharmaceutical Sciences, Graduate School, Kyung Hee University, Seoul 02447, Korea; 3 Department of New Material Development, COSMAXBIO, Seongnam 13486, Korea; ykshin@cosmax.com (Y.-K.S.); jsyu@bu.edu (J.-S.Y.); 4 Clinical Research Center of H PLUS Yangji Hospital, Sillim-dong, Gwanak-gu, Seoul 08779, Korea; juspa@naver.com

**Keywords:** *Hydrangea serrata* (Thunb.) Ser., obesity, body weight, body fat, clinical trial

## Abstract

Obesity is a major health problem that is caused by body fat accumulation and that can lead to metabolic diseases. Owing to several side effects of the currently used antiobesity drugs, natural plants have risen as safe and potential candidates to alleviate obesity. We have previously reported the antiobesity effect of *Hydrangea serrata* (Thunb.) Ser. leaves extract (WHS) and its underlying mechanisms. As an extension of our preclinical studies, this study aimed to investigate the effect of WHS on body weight and body fat reduction in overweight or obese humans. A total of 93 healthy overweight or obese males and females, aged 19–65 years, with body mass indexes (BMIs) ≥ 25 and <32 kg/m^2^, were recruited and received either an oral administration of 600 mg of WHS, or placebo tablets for 12 weeks. Daily supplementation with WHS decreased body weights, body fat masses, and BMIs compared with the placebo-treated group. The hip circumferences, visceral fat areas, abdominal fat areas, and visceral-to-subcutaneous ratios decreased after WHS supplementation. No significant side effects were observed during or after the 12 weeks of WHS intake. In conclusion, WHS, which has beneficial effects on body weight and body fat reduction, could be a promising antiobesity supplement that does not produce any side effects.

## 1. Introduction

Overweight and obesity are emerging health problems that can occur across ages, sexes, and races. The etiology of obesity is highly complex and includes genetic, physiological, environmental, social, and economic factors, in addition to a high-energy intake relative to the energy expenditure, which is the most common cause of obesity [1]. It is not only a cosmetic problem but also a health threat that increases the occurrence of metabolic diseases, such as type 2 diabetes mellitus (T2DM), cardiovascular disease (CVD), hyperlipidemia, fatty liver disease, and several types of cancers [2,3].

The degree of obesity is categorized by the body mass index (BMI), which is defined as a 18 ≤ BMI < 25 kg/m^2^ as the normal range, a 25 ≤ BMI < 30 kg/m^2^ as the overweight range, and a BMI ≥ 30 kg/m^2^ as the obese range [4]. Obese people often need to be medicated to manage their weight and excess body fat accumulation. Current agents for weight loss exert their effects by regulating appetite and fat absorption from the gut. As appetite suppressants, such as diethyl propion, fluoxetine, and lorcaserin, generally affect the central nervous system (CNS), they can induce other CNS effects, such as nervousness, insomnia, anxiety, and depression [5]. Xenical (orlistat), which blocks fat digestion and absorption by inhibiting lipase activity, can have gastrointestinal side effects, including steatorrhea and diarrhea. Therefore, as the global market for nutraceuticals grows, numerous bioactive materials from natural sources have been investigated and developed as health supplements to mitigate obesity without severe side effects [6]. For example, *Cissus quadrangularis* extract inhibited lipid accumulation by downregulating the expression of adipogenesis/lipogenesis-related proteins [7]. Moro orange juice intake reduced body weight and lipid accumulation in both obese mice and humans [8,9]. Epigallocatechin-3-gallate from green tea has the potential to increase fat oxidation and thermogenesis [10,11]. Moreover, astaxanthin, a marine carotenoid that possesses antioxidant activity, prevented the pathological remodeling of adipose tissue and stimulated mitochondrial biogenesis in muscle [12,13].

Hydrangea leaves extract is included in the list of GRAS (Generally Recognized as Safe Substance), and is therefore recognized as safe for eating or drinking as foods. It is widely consumed as a form of herbal tea, named “Gamrocha” or “Amacha”, meaning “sweet taste” in Korea and Japan [14]. We previously reported the potential of the hot water extract of *Hydrangea serrata* (Thunb.) Ser. leaves (WHS) to reduce body weight and body fat weight [15,16]. AMP-activated protein kinase (AMPK) is a crucial intracellular energy sensor that regulates energy homeostasis in various tissues, such as the liver, skeletal muscle, and adipose tissue [17]. In white adipose tissue (WAT), AMPK regulates lipogenesis and lipolysis by decreasing fatty acid (FA) uptake and increasing FA oxidation [18]. AMPK also modulates the thermogenesis by activating PGC-1α in brown adipose tissue (BAT), which dissipates energy as heat [19]. That is to say, AMPK can be a major molecular target for alleviating obesity by regulating both adipogenesis and thermogenesis. We found that the oral administration of WHS exhibits an antiobesity effect by regulating both adipogenesis and thermogenesis by targeting AMPK activation in obese mice [15,16]. As an extension of our preclinical studies, we investigated whether WHS exerts a potent inhibitory effect on body weight and body fat weight in overweight or obese humans, and discovered promise in the development of WHS as a health supplement for ameliorating obesity.

## 2. Materials and Methods

### 2.1. Test Material

A hot water extract from the leaves of *H. serrata* was prepared in the form of a tablet. Test tablets (600 mg WHS) and placebo tablets were prepared, as described previously [20]. The placebo tablets used in this study were indistinguishable from the test tablets.

### 2.2. Subjects

**Figure 1:**
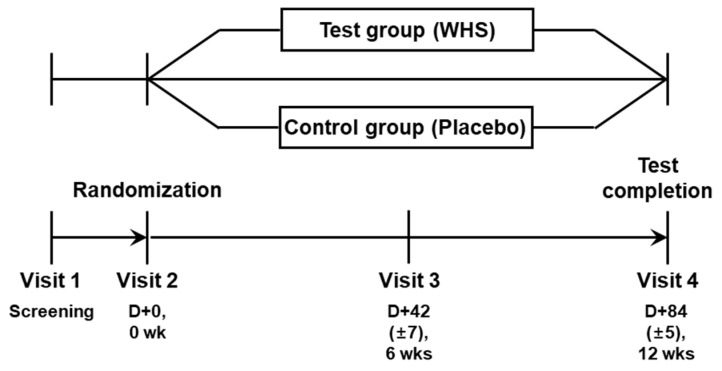
Process of study.

Healthy males and females, aged 19–65 years, with BMIs from 25 to <32 kg/m^2^ at Visit 1 and Visit 2 were recruited for this study (Figure 1). The subjects were informed of the objective and protocol of the study, and the foreseeable risks involved in the trial. All participants voluntarily signed a written informed consent form. The subjects were evaluated for eligibility according to the exclusion criteria, as follows: (1) Those who are receiving treatment for serious diseases (e.g., cardiovascular, immunological, respiratory, hepatobiliary, renal, neurological, musculoskeletal, mental, and infectious diseases, or malignant tumor); (2) Those who have taken any bariatric drugs for losing weight (e.g., anorexiants, fat absorption inhibitors, GLP-1 receptor agonists), psychiatric drugs for depression and schizophrenia, β-blockers, diuretics, contraceptives, steroids, female hormonal injections, or additional health supplements for weight control within one month of Visit 1; (3) Uncontrolled hypertensive patients who have a 160/100 mmHg or higher blood pressure after a 10-min rest; (4) Those whose fasting plasma glucose levels are 126 mg/dL or higher, or diabetic patients taking antidiabetic drugs (insulin, hypoglycemic agents); (5) Those whose TSH levels are ≤0.1 μIU/mL or ≥10 μIU/mL; (6) Those whose creatinine levels are two times higher than the normal standard; (7) Those whose AST (GOT) or ALT (GPT) levels are three times higher than the normal standard; (8) Those who have severe gastrointestinal disorder; (9) Those who have been hospitalized, treated with drugs, or rehabilitated because of alcohol use, alcohol-induced disorders, heart disease, or central nervous system disorders; (10) Those who suffer from musculoskeletal disorder; (11) Those who have had a weight change of 10% or more within three months of Visit 1; (12) Those who have participated in a commercial obesity program within three months of Visit 1; (13) Those who have participated or plan to participate in other interventional clinical trials (including human clinical trials) within three months of Visit 1; (14) Women who had undergone, or planned to undergo, pregnancy or nursing during the trials; (15) Those who are sensitive or allergic to ingredients included in the test formulation; (16) Those who are considered to be inappropriate to participate in the study by the investigator.

### 2.3. Study Design

The study design is illustrated in Figure 1. Before proceeding with this randomized double-blind placebo-controlled clinical study, it was determined whether the participants met the inclusion/exclusion criteria through a screening visit (Visit 1). A total of 189 applicants were enrolled, and 120 subjects were selected, and were randomly assigned to the control (placebo) and test (WHS) groups in a 1:1 ratio at Visit 2 (60 participants were allocated to each group). The participants were instructed to take a 600-mg WHS or placebo tablet with water once daily for 12 weeks. The study protocol was approved by the Institutional Review Board of H Plus Yangji Hospital (HYJ 2020-04-003-014). This trial was registered at the Clinical Research Information Service (CRiS), Republic of Korea (KCT0005594, https://cris.nih.go.kr/cris/search/detailSearch.do/18021 accessed on 1 December 2021).

### 2.4. Sample Size Determination and Power Calculation

We expected the clinically significant change in our primary outcome, the body fat mass, on the basis of the previously reported study that is the same as our trial. The changes in the body fat masses from baseline were −2.4 ± 1.7 kg and −3.8 ± 2.4 kg in the placebo and the test groups, respectively, and the pooled standard deviation (SD) value is 2.07 (σ) [21]. We set the difference in the body fat mass between the placebo and the test groups (Δ) conservatively, as −1.23. We estimated the sample size using a superiority test and a two-sided test, and considering type 1 (α) and type 2 (β) errors of 0.05 and 0.2 with an 80% power, respectively. The sample size was calculated using the equation as follows: *n* = {(Z_α/2_ + Z_β_)^2^ × σ^2^ × 2}/Δ^2^. The Z_α/2_ and Z_β_ signify the critical values that the areas of the right tail become at α/2 and β, respectively. On the basis of the above equation, the minimum sample size is calculated as *n* = 45 per group in this study. Considering the 25% drop-out rate, we determined a sample size of *n* = 60 per group.

### 2.5. Clinical Outcomes

The primary efficacy outcome was a reduction in the body fat mass from baseline to 12 weeks (Visit 4), after randomization. The body fat mass was analyzed using dual-energy X-ray absorptiometry (DEXA). The secondary efficacy outcomes included changes in the body weight, the waist and hip circumferences, the waist-to-hip ratio (WHR), the body mass index (BMI), the body fat percentage, the visceral and subcutaneous fat areas, the total abdominal fat area, the visceral-to-subcutaneous ratio (VSR), blood lipids (e.g., total cholesterol, high-density lipoprotein (HDL)-cholesterol, low-density lipoprotein (LDL)-cholesterol, and triglyceride (TG)), adiponectin, and leptin. The visceral and subcutaneous fat areas and the total abdominal fat area were assessed from the space of the L4–L5 intervertebral, using a computerized tomography (CT) scanner.

The safety was assessed by monitoring all of the adverse events and results from the blood chemical tests, including aspartate aminotransferase (AST), alanine aminotransferase (ALT), and γ-glutamyl transpeptidase (γ-GTP) for the liver function tests, blood urea nitrogen (BUN) and creatinine for the renal function tests, and high sensitive C-reactive protein (hs-CRP) for the inflammatory status. The vital signs (blood pressure and pulse) of the subjects were measured.

### 2.6. Statistical Analysis

Statistical analyses were performed using the SAS^®^ system (Version 9.4, SAS Institute, Cary, NC, USA). The full analysis and per-protocol datasets were used for the statistical analyses. The significant differences in the baseline demographic characteristics were analyzed using the Chi-square test, the Fisher’s exact test, or the Wilcoxon rank-sum test. The significance of the differences in the changes before and after intake (compared within groups) was analyzed using a paired *t*-test, and the differences in the degree of changes between the groups were analyzed by performing a two-sample *t*-test or Wilcoxon rank-sum test. In addition, a generalized linear model (GLM) was conducted with the variables, gender, age, drinking, smoking, and exercise, as co-variates. It was planned that the GLM was to be conducted if there was any significant difference between the base characteristic groups, but there was no statistically significant difference among the base characteristic groups. The data are presented as mean ± standard deviation (SD). The differences were considered statistically significant at a *p*-value < 0.05.

## 3. Results

### 3.1. Baseline Characteristics of Subjects

**Figure 2:**
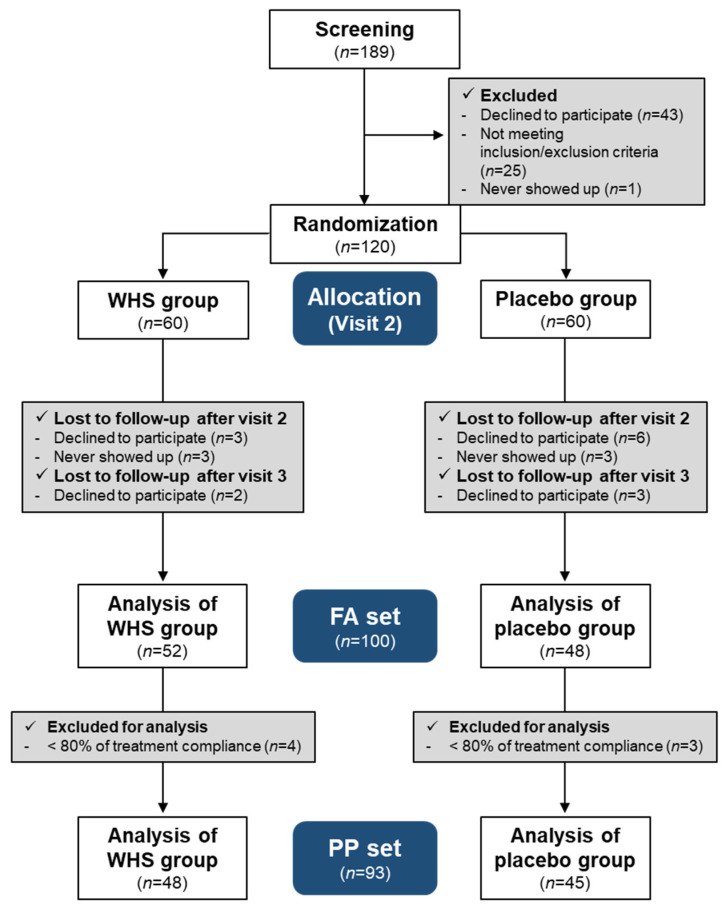
Flow diagram of the enrolled participants.

Of the 189 volunteers, 120 participants who met the criteria were randomly allocated to the test and placebo groups and were administered the WHS and placebo tablets, respectively. Five participants from the WHS group and nine from the placebo group withdrew from the study. Three participants each from the WHS and placebo groups did not appear in the trial. Therefore, 52 and 48 participants administered the WHS and placebo, respectively, completing the study (Figure 2). Participants with less than 80% compliance were excluded from the per-protocol set analysis. The demographic characteristics of the participants are tabulated in Table 1. There were no significant differences in the general baseline characteristics of the subjects, such as sex, age, drinking, and smoking, between the two groups.

### 3.2. Effect of WHS on the Reduction of Fat Mass and Fat Percentage

**Figure 3:**
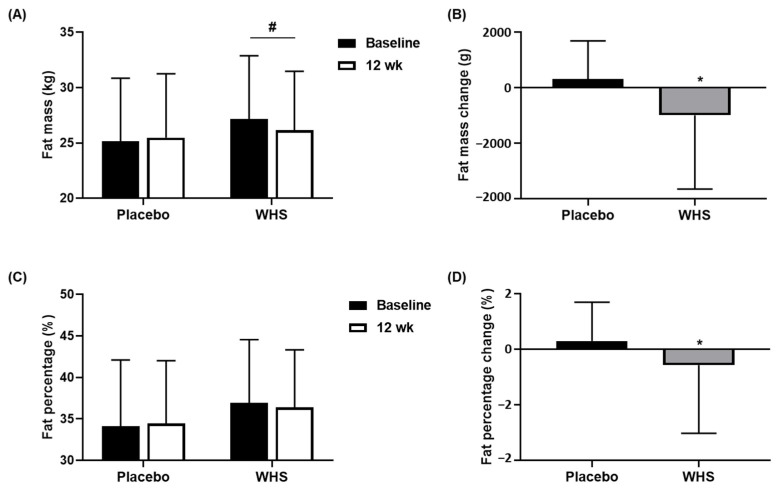
Changes in body fat masses and fat percentages. Changes in (**A**,**B**) fat mass and (**C**,**D**) fat percentage were measured in placebo- and WHS-treated groups at baseline and 12 weeks. Values are present as mean ± SD; ^#^ *p* < 0.05 derived from paired *t*-test within groups (Weeks 0 vs. 12), and ^*^ *p* < 0.05 derived from a two-sample *t*-test or Wilcoxon rank-sum test between groups (Placebo (*n* = 45) vs. WHS (*n* = 48)).

As the primary outcome for the efficacy evaluation, the changes in the body fat masses of the subjects were determined through DEXA scans. As shown in Figure 3A,B, the body fat masses were significantly decreased when the WHS was administered for 12 weeks (*p* = 0.0130), which contrastively increased in the subjects treated with the placebo. The change in the body fat mass of the WHS group decreased by 991.71 ± 2660.98 g, whereas that of the placebo group increased by 312.91 ± 1379.49 g, after 12 weeks of oral intake, which showed statistically significant differences between the two groups (*p* = 0.0049). Although there were no significant differences before and after the experiment within the groups, we observed a significant difference in the change in the fat percentage between the two groups (*p* = 0.0467, Figure 3C,D). The body fat percentage in the WHS group was reduced by 0.57 ± 2.46%, whereas it was increased in the placebo group by 0.31 ± 1.39% from the baseline. The maximum changes in the body fat masses and fat percentages were −6504.00 g and −6.80%, respectively, after 12 weeks of WHS administration.

### 3.3. Effect of WHS on Body Weight and BMI

**Figure 4:**
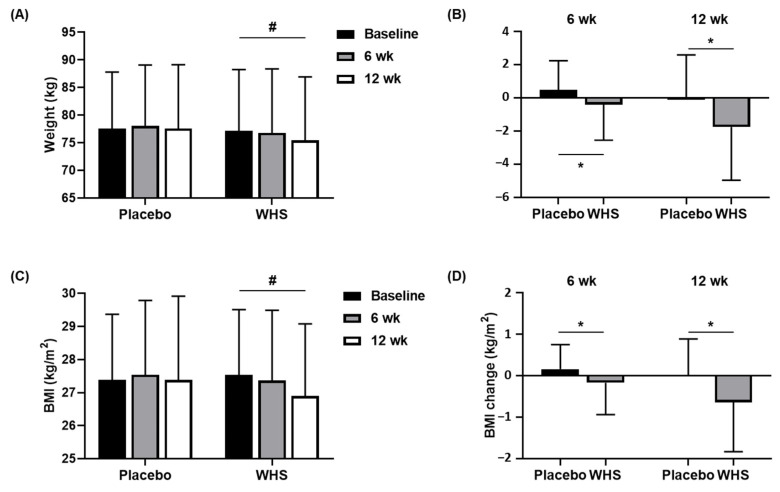
Changes in body weights and BMIs. Changes in (**A**,**B**) weight and (**C**,**D**) BMI were measured in placebo- and WHS-treated groups at baseline, 6, and 12 weeks. Values are present as mean ± SD; ^#^ *p* < 0.05 derived from paired *t*-test within groups (Weeks 0 vs. 12), and ^*^ *p* < 0.05 derived from a two-sample *t*-test or Wilcoxon rank-sum test between groups (Placebo (*n* = 45) vs. WHS (*n* = 48)).

The body weights and BMIs of all the subjects were measured at Visits 3 and 4 (after 6 weeks and 12 weeks of oral intake, respectively). The body weights of the WHS group gradually decreased by 0.40 ± 2.15 kg (*p* = 0.2015) and 1.75 ± 3.21 (*p* = 0.0004) kg after 6 and 12 weeks, respectively (Figure 4A); however, there was no significant difference in the body weights of the placebo group. As shown in Figure 4B, the mean changes in the body weights between the two groups showed significant differences at both Visits 3 and 4 (*p* = 0.0480 and *p* = 0.0020, respectively). The maximum changes in the body weights of the WHS group were −5.60 kg and −11.90 kg after 6 and 12 weeks, respectively. The BMIs of the WHS group also reduced by 0.17 ± 0.77 kg/m^2^ (*p* = 0.1371) and 0.64 ± 1.19 kg/m^2^ (*p* = 0.0005) after 6 and 12 weeks, respectively (Figure 4C), which showed significant differences compared with the placebo group at Visits 3 and 4 (*p* = 0.0433 and *p* = 0.0026, respectively, Figure 4D). The maximum changes in the BMIs were −2.30 kg/m^2^ and −4.90 kg/m^2^, after 6 and 12 weeks of WHS intake, respectively.

### 3.4. Effect of WHS on the Waist and Hip Circumferences

**Figure 5:**
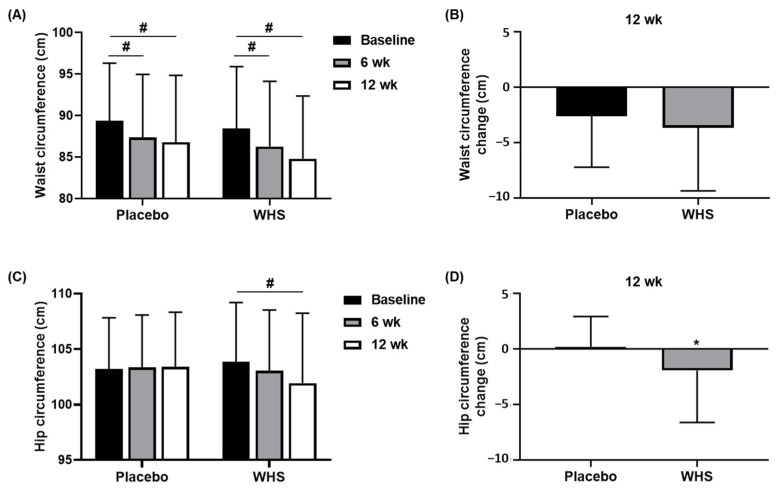
Changes in waist and hip circumferences. Changes in (**A**,**B**) waist circumference and (**C**,**D**) hip circumference were measured in placebo- and WHS-treated groups at baseline, 6, and 12 weeks. Values are present as mean ± SD; ^#^ *p* < 0.05 derived from paired *t*-test within groups (Weeks 0 vs. 6 or 12), and ^*^ *p* < 0.05 derived from a two-sample *t*-test or Wilcoxon rank-sum test between groups (Placebo (*n* = 45) vs. WHS (*n* = 48)).

As shown in Figure 5A, the waist circumferences in both groups had significantly decreased after 6 weeks (*p* = 0.0111 in the placebo group, and *p* = 0.0023 in the WHS group) and 12 weeks of intake (*p* = 0.0005 in the placebo group, and *p* < 0.0001 in the WHS group). Although the reduction in the waist circumferences was greater in the WHS group (−3.65 ± 5.71 cm) than in the placebo group (−2.59 ± 4.63 cm), there was no significant difference between the two groups (Figure 5B). The hip circumference of the WHS group was gradually decreased during the experiment, whereas no such change was observed in the placebo group (Figure 5C). There were significant differences in the hip circumferences between the two groups after 12 weeks (Figure 5D).

### 3.5. Effect of WHS on Body Fat Area

**Figure 6:**
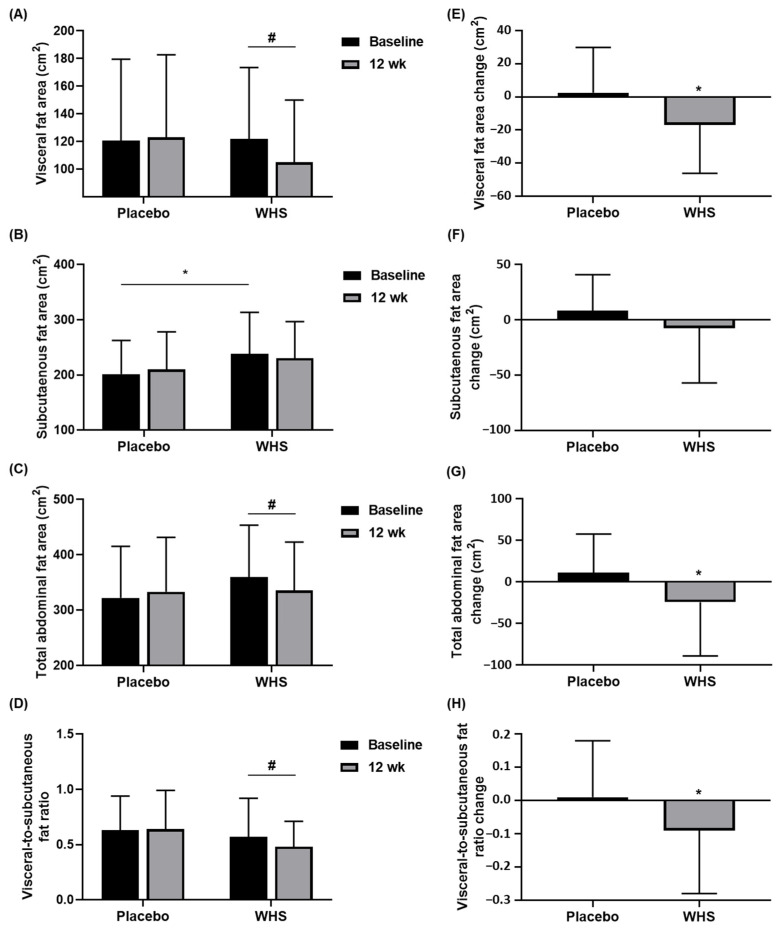
Changes in body fat areas. (**A**) Visceral fat area, (**B**) subcutaneous fat area, (**C**) total abdominal fat area, and (**D**) visceral-to-subcutaneous fat ratio were measured in placebo- and WHS-treated groups at baseline and 12 weeks. (**E**–**H**) Changes in these parameters between Weeks 0 and 12. Values are present as mean ± SD; ^#^ *p* < 0.05 derived from paired *t*-test within groups (Weeks 0 vs. 12), and ^*^ *p* < 0.05 derived from a two-sample *t*-test or Wilcoxon rank-sum test between groups (Placebo (*n* = 45) vs. WHS (*n* = 48)).

CT scans were performed to analyze the abdominal fat areas. According to the within-group analyses, the visceral fat areas (VFAs), the total abdominal fat areas, and the visceral-to-subcutaneous fat ratios (VSRs), but not the SFAs, had significantly decreased after 12 weeks of WHS ingestion, whereas the placebo-treated groups showed no significant differences in these parameters (Figure 6A–D). The maximum changes in the VFA, SFA, total abdominal fat area, and VSR were about two times larger in the WHS group (−101.11 cm^2^, −160.87 cm^2^, −223.02 cm^2^, and −1.01, respectively) than in the placebo group (−53.18 cm^2^, −85.94 cm^2^, −115.87 cm^2^, and −0.39, respectively). Significant differences were also observed between the two groups in the changes in these parameters from the baseline, except for the SFAs (Figure 6E–H).

### 3.6. Effect of WHS on the Plasma Levels of Lipid Metabolism Markers and Adipokines

The plasma levels of the total cholesterol, HDL, LDL, and TG were analyzed at the start and at the end of the experiment. As shown in Table 2, all of the parameters, except for the total cholesterol, tended to decrease in both the placebo and WHS groups. The plasma LDL levels of the placebo-treated subjects and the plasma TG levels of the WHS-treated subjects were significantly reduced after 12 weeks. However, there were no significant differences in these parameters between the two groups. Table 3 shows the levels of plasma adipokines, including adiponectin and leptin. According to the intergroup comparison, the adiponectin levels decreased in the WHS group and, significantly decreased in the placebo group. The leptin levels in the WHS group tended to decrease, while those in the placebo group remained almost unchanged; however, these changes did not show any significant differences within and between the groups.

### 3.7. Safety Evaluation of WHS

The plasma levels of AST, ALT, γ-GTP, creatinine, BUN, and hs-CRP, and the resting blood pressure and pulse rates were measured to evaluate the safety of WHS (Table 4). These parameters were within the normal ranges and did not change significantly, indicating that the daily intake of WHS for 12 weeks did not cause any side effects.

## 4. Discussion

The adipose tissues are mainly divided into two depots: subcutaneous adipose tissue (SAT), and visceral adipose tissue (VAT). Emerging evidence suggests that the overloaded fat in SAT, which reaches its storage limit, causes ectopic fat accumulation at undesired sites, including at the heart, liver, muscle, and VAT [22]. As excessive ectopic fat induces regional inflammation and insulin resistance by secreting proinflammatory cytokines and adipokines, VAT is believed to be more strongly associated with the development of metabolic diseases, such as CVD and T2DM, compared to SAT [23]. In addition to the absolute fat mass or area, the VSR is a metric of the body fat distribution that is correlated with cardiometabolic risk: the higher it is, the higher the risk for CVD [24]. In this study, the oral ingestion of WHS significantly reduced VFA and VSR compared with the placebo-treated group. These results suggest that WHS could not only ameliorate body fat accumulation, but also prevent the progression of obesity-induced metabolic syndromes. Analyzing the correlations between the fat mass and the parameters related to the metabolic syndromes, including the insulin sensitivity and glycemia of WHS, could support this assumption.

Leptin is a hormone that is primarily secreted by adipocytes; therefore, circulating leptin levels directly reflect the fat mass stored in adipose tissue [25]. We previously found that WHS inhibits plasma leptin levels in obese mice [15]. However, the plasma leptin levels showed no significant difference between the placebo- and WHS-administrated groups in this study. Considering the different lifespans of mice and humans [26], we expected that WHS could significantly inhibit the plasma leptin levels with long-term supplementation. In our preclinical studies, WHS was found to reduce the total cholesterol, and the LDL and TG levels in mouse plasma [15,16]. However, these lipid profiles were almost unchanged or decreased in both the placebo- and WHS-administrated groups in this study, with no statistical differences between the groups. As the subjects recruited in the present study were overweight or obese, but otherwise healthy, without any signs of metabolic syndromes, these parameters were within the normal range and the changes were not significant.

Although various botanical sources have been reported to have antiobesity activities in preclinical studies, some of them, including *Garcinia cambogia* extract [27,28], green tea extract [29], and glucomannan [30], have failed to promote weight loss in humans. Instead, mixed plant extracts containing *Garcinia cambogia*, *Coffea Arabica*, and green tea have shown a significant weight loss effect, which is similar to that of WHS alone [31,32]. Although Puer tea extract, *Ephedra sinica* extract, and Yerba Mate each exhibited their inhibitory effects on the body weight and body fat mass, the doses were somewhat high (3 g/day, 4 g/day, and 3 g/day, respectively) [33,34,35]. It is noteworthy that WHS showed comparable antiobesity potential at a relatively low dose (600 mg) in this study. In our previous studies, we identified the chemical constituents of the extract of *Hydrangea serrata* leaves and identified hydrangenol as an active compound of WHS concerning the antiphotoaging activities [36,37,38]. Likewise, we expected the antiobesity potential of hydrangenol and are planning to investigate it.

During the clinical trials, the symptom severities of the adverse reactions that occurred in the WHS group were nine mild and eight moderate cases. With regard to the relevance to the test food for human application, two cases were declared “possibly related”, while fourteen cases were declared “possibly unrelated”, and one case was declared “definitely unrelated” by the investigator. The two abnormal reactions possibly related to WHS included menstrual pain and stomach pain, which were treated with the drugs and found to be completely cured. Although the causality relationship between the adverse reactions and the test product needs to be determined, it seems that these side effects are rare and negligible.

## 5. Conclusions

The present study demonstrated the efficacy and safety of WHS in overweight or obese humans. A daily intake of 600 mg of WHS for 12 weeks significantly decreased body weight and fat mass, with a reduction in the BMI, body fat percentage, hip circumference, VFA, total abdominal fat area, and VSR. To fully determine the efficacy and possible side effects of WHS, a larger sample size with a longer application is needed. With this evidence, the development of WHS as a potential weight loss supplement is promising.

## Figures and Tables

**Table 1:** Baseline demographic characteristics of the subjects.

Variable	Placebo (*n* = 45)	WHS (*n* = 48)	*p*-Value
Sex (M/F)	26/19	20/28	0.1204 ^1^
Age	30.47 ± 9.62	32.71 ± 10.98	0.3189 ^2^
Drinker (Y/N)	25/20	26/22	0.9363 ^3^
Smoker (Y/N)	35/10	33/15	0.6810 ^3^

Values are present as mean ± SD; *p*-values are analyzed by ^1^ Chi-square test, ^2^ Wilcoxon rank-sum test, or ^3^ Fisher’s exact test. M/F; Male/Female, Y/N; Yes/No.

**Table 2:** Lipid profiles of subjects.

Variable	Period	Placebo (*n* = 45)	WHS (*n* = 48)	*p*-Value
Total cholesterol (mg/dL)	Week 0	196.80 ± 34.35	196.44 ± 34.70	
	Week 12	192.31 ± 26.88	198.00 ± 28.72	
	Δ (0–12)	−4.49 ± 21.12	1.56 ± 23.12	0.1917 ^1^
HDL-cholesterol (mg/dL)	Week 0	55.69 ± 12.61	58.52 ± 12.22	
	Week 12	53.84 ± 10.41	56.73 ± 11.78	
	Δ (0–12)	−1.84 ± 7.56	−1.79 ± 10.20	0.8716 ^2^
LDL-cholesterol (mg/dL)	Week 0	128.67 ± 29.42	129.04 ± 31.48	
	Week 12	118.80 ± 23.19 ^#^	123.54 ± 25.39	
	Δ (0–12)	−9.87 ± 21.14	−5.50 ± 25.75	0.3755 ^1^
Triglyceride (mg/dL)	Week 0	138.73 ± 112.89	115.19 ± 71.28	
	Week 12	124.24 ± 112.77	90.10 ± 52.55 ^#^	
	Δ (0–12)	−14.49 ± 95.13	−25.08 ± 63.71	0.3934 ^2^

Values are present as mean ± SD; *p*-values were analyzed by a ^1^ two-sample *t*-test or ^2^ Wilcoxon rank-sum test between groups (Placebo vs. WHS). ^#^ *p* < 0.05, derived from paired *t*-test within groups (Weeks 0 vs. 12).

**Table 3:** Plasma levels of adipokines in subjects.

Variable	Period	Placebo (*n* = 45)	WHS (*n* = 48)	*p*-Value ^1^
Adiponectin (μg/mL)	Week 0	5.43 ± 1.94	6.18 ± 2.85	
	Week 12	4.88 ± 1.72 ^#^	6.00 ± 2.46	
	Δ (0–12)	−0.55 ± 0.98	−0.18 ± 1.92	0.1232
Leptin (ng/mL)	Week 0	10.20 ± 8.84	11.30 ± 7.63	
	Week 12	10.19 ± 8.77	10.09 ± 8.07	
	Δ (0–12)	−0.01 ± 4.96	−1.21 ± 5.73	0.4675

Values are present as mean ± SD; ^1^ *p*-values were analyzed by Wilcoxon rank-sum test between groups (Placebo vs. WHS). ^#^ *p* < 0.05 derived from a paired *t*-test within groups (Weeks 0 vs. 12).

**Table 4:** Safety analyses.

Variable	Period	Placebo (*n* = 45)	WHS (*n* = 48)	*p*-Value
AST (U/L)	Week 0	23.85 ± 9.74	24.25 ± 8.52	
	Week 12	24.44 ± 9.49	29.62 ± 24.50	
	Δ (0–12)	0.17 ± 7.32	5.04 ± 25.05	0.8791 ^2^
ALT (U/L)	Week 0	25.43 ± 16.79	26.60 ± 15.72	
	Week 12	24.33 ± 18.41	34.54 ± 64.83	
	Δ (0–12)	−0.52 ± 9.34	8.10 ± 63.32	0.7455 ^2^
γ-GTP (U/L)	Week 0	28.78 ± 18.66	27.13 ± 18.27	
	Week 12	29.42 ± 26.28	24.63 ± 19.21	
	Δ (0–12)	0.77 ± 13.26	−2.17 ± 7.95	0.1433 ^2^
Creatinine (mg/dL)	Week 0	0.73 ± 0.16	0.70 ± 0.15	
	Week 12	0.77 ± 0.19	0.73 ± 0.16	
	Δ (0–12)	0.03 ± 0.09	0.03 ± 0.06	0.9558 ^1^
BUN (mg/dL)	Week 0	12.57 ± 3.51	12.38 ± 2.79	
	Week 12	12.57 ± 3.89	12.52 ± 3.57	
	Δ (0–12)	0.03 ± 3.07	0.18 ± 3.54	0.8495 ^2^
hs-CRP (mg/L)	Week 0	2.48 ± 6.21	1.09 ± 1.09	
	Week 12	1.59 ± 2.88	1.22 ± 1.49	
	Δ (0–12)	−0.90 ± 4.56	0.14 ± 0.86	0.7986 ^2^
Systolic blood pressure (mmHg)	Week 0	130.22 ± 12.53	128.00 ± 11.42	
	Week 12	124.83 ± 13.14	123.46 ± 12.45	
	Δ (0–12)	−6.60 ± 14.01	−4.98 ± 12.55	0.4538 ^2^
Diastolic blood pressure (mmHg)	Week 0	78.88 ± 10.48	78.12 ± 9.98	
	Week 12	75.50 ± 11.12	75.25 ± 9.52	
	Δ (0–12)	−4.71 ± 11.35	−2.73 ± 8.61	0.3266 ^1^
Pulse (beats/min)	Week 0	83.03 ± 11.85	84.15 ± 11.62	
	Week 12	80.81 ± 13.36	81.02 ± 11.78	
	Δ (0–12)	−3.48 ± 12.71	−3.04 ± 12.14	0.8596 ^1^

Values are present as mean ± SD; *p*-values were analyzed by a ^1^ two-sample *t*-test, or ^2^ Wilcoxon rank-sum test between groups.
